# Iron-specific Signal Separation from within Heavy Metal Stained Biological Samples Using X-Ray Microtomography with Polychromatic Source and Energy-Integrating Detectors

**DOI:** 10.1038/s41598-018-25099-z

**Published:** 2018-05-15

**Authors:** Tsvi Katchalski, Tom Case, Keun-young Kim, Ranjan Ramachandra, Eric A. Bushong, Thomas J. Deerinck, Matthias G. Haberl, Mason R. Mackey, Steven Peltier, Guillaume A. Castillon, Nobuko Fujikawa, Albert R. Lawrence, Mark H. Ellisman

**Affiliations:** 1 National Center for Microscopy and Imaging Research (NCMIR), University of California San Diego, 9500 Gilman Dr. MC 0608, La Jolla, CA 92093-0608 USA; 2 Carl Zeiss X-Ray Microscopy, 4385 Hopyard Road, Suite 100, Pleasanton, CA 94588 USA; 3 Departments of Neurosciences and Bioengineering, University of California San Diego, 9500 Gilman Dr. MC 0608, La Jolla, CA 92093-0608, USA

## Abstract

Biological samples are frequently stained with heavy metals in preparation for examining the macro, micro and ultra-structure using X-ray microtomography and electron microscopy. A single X-ray microtomography scan reveals detailed 3D structure based on staining density, yet it lacks both material composition and functional information. Using a commercially available polychromatic X-ray source, energy integrating detectors and a two-scan configuration labelled by their energy- “High” and “Low”, we demonstrate how a specific element, here shown with iron, can be detected from a mixture with other heavy metals. With proper selection of scan configuration, achieving strong overlap of source characteristic emission lines and iron K-edge absorption, iron absorption was enhanced enabling K-edge imaging. Specifically, iron images were obtained by scatter plot material analysis, after selecting specific regions within scatter plots generated from the “High” and “Low” scans. Using this method, we identified iron rich regions associated with an iron staining reaction that marks the nodes of Ranvier along nerve axons within mouse spinal roots, also stained with osmium metal commonly used for electron microscopy.

## Introduction

X-ray microtomography (XRM) has established itself as a powerful imaging method for biological samples. The high penetration of X-rays makes it attractive for whole body/organ imaging, generating 3D volumes and imaging optically opaque samples while exposing the samples to minimal radiation damage^1,2^. It has also been shown to assist in selecting regions of interest (ROI) prior to performing electron microscopy (EM), improving workflow efficiency^3,4^. Standard XRM with lab-based instruments can now provide isotropic resolution near 0.5 μm.

In general, reconstructed images will display overall attenuation correlated with specimen density without the ability to discriminate variations in material composition or the ability to detect a specific marker. This generally has little impact given that the staining methods, in many cases, are not very specific. However, we reasoned that having the ability to follow a specific marker would provide an advantage for biological specimens, especially if it could be linked to light microscopic (LM) probe-based methods that allow dynamic imaging in a fluorescence microscope. Numerous probes have been developed that allow one to convert fluorescent signals to electron-dense metallic labels, thereby allowing correlated LM and EM imaging of specimens^5–9^. As investigators are now turning to XRM as a means of tracking ROI from LM to EM, there is a potential demand for a means to track specific metals by XRM^3,4,10–14^.

Various strategies have been developed for performing material analysis using X-rays. These include methods using monochromatic X-ray sources either from large synchrotrons^15^ or laser produced plasma^16^, and recently a method using a polychromatic source and Ross filter pairs^17^. While the former methods involve using specialized sources, the latter has the advantage of using accessible (laboratory scale) X-ray tubes. On the other hand, the Ross filter pair involves taking four measurements – one for each filter, generating effectively two narrow band-pass filters resulting in relatively low source power utilization. In essence, all the above referenced methods present some sort of K-edge imaging principles^18^ whereby the element of interest is typically imaged with energy just below and just above the K-edge, using the large abrupt variation in absorption to generate high contrast. Other methods include dual energy as used in medical imaging, where the different contributions of photoelectric and Compton scatter to the total absorption are used (e.g., to separate calcium or bone from iodine^19^). Typically, in those cases the minimum X-ray energy of substantial power due to filtration is considerably above any low Z-element K-edge energy. Also it is worth noting that this method will be further enhanced by employing next generation photon counting detectors^20–22^, now being deployed on a growing number of systems.

In the work presented here we used XRM to detect specifically stained iron regions of micron and sub-micron scale in a biological sample stained with other heavy metals typically used for electron microscopy staining, such as osmium. We used a two-scan configuration and scatter plots to perform material analysis. The first scan uses the characteristic emission lines of the tungsten source to enhance the absorption of iron, which has a strong K-edge onset at an energy just smaller than the first in a series of emission lines. The second scan is performed after X-pray filtration which absorbs strongly the characteristic emission lines relative to the higher energy continuum part of the spectrum causing a reduction in iron absorption. We initially demonstrated results on a phantom, then theoretically explored optimal scan configuration and finally applied this information to identify iron rich regions associated with the nodes of Ranvier in samples of mouse spinal root stained with iron and osmium. These results could potentially allow following iron specific biological markers such as ferritin^23^ and thus, introduce functionality into XRM measurements.

## Results and Discussion

**Figure 1:**
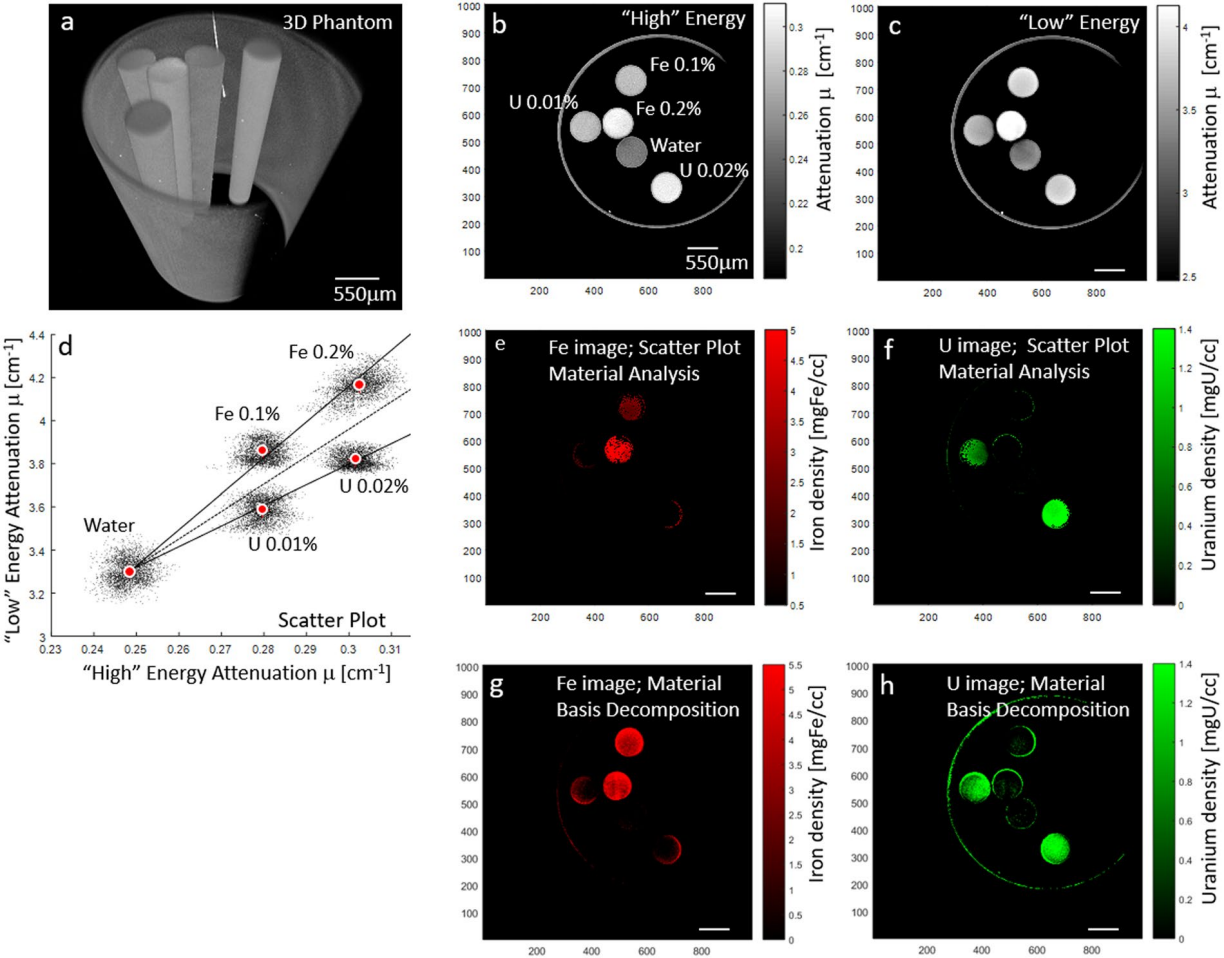
“High” and “Low” scan configuration of a phantom containing uranium and iron aqueous solutions in microtubes. Material analysis is performed in the middle row by scatter plot material analysis and in the last row by material basis decomposition. (**a**) 3D volume rendering of phantom configuration. (**b**,**c**) “High” and “Low” scan slice image, at 60 kVp with 25 μm thick iron filter and 30 kVp without filtration respectively. Material concentration in [%] by weight of compound, ferric chloride or uranyl acetate, are marked in (**b**). Water filled microtube and air were used to calibrate the results to give values in attenuation units [cm^−1^]. (**d**) Scatter plot of “Low” vs. “High” pixel values showing “clouds” of distinct chemical composition. (**e**,**f**) Scatter plot material analysis of iron and uranium using (**d**), which after calibration result in density images in units of [mgFe/cc.] and [mgU/cc] for iron and uranium respectively. (**g**,**h**) Material basis decomposition results for iron and uranium.

Initially, we tested feasibility using a phantom containing microtubes holding either aqueous iron, aqueous uranium, or pure water, as shown in Fig. 1. Uranium was used to represent the heavy metal stains typically used in biological staining, which can include osmium (^76^Os), lead (^82^Pb), and uranium (^92^U). Uranium was chosen over osmium tetroxide (OsO_4_) as it is considerably less toxic. Iron (^26^Fe) was the target metal of interest. The exact phantom composition is detailed in supplemental material S1. The microtubes contained aqueous ferric chloride (FeCl_3_·6H_2_O) at 0.2% and 0.1% by weight or uranyl acetate, UA (UO_2_(CH_3_COO)_2_·2H_2_O) at 0.02% and 0.01% by weight. A pure water tube was included for calibration. A reconstructed 3D volume rendering of the phantom is shown in Fig. 1(a). Slice images from the two scans performed at 60kVp with 25um-thick iron filter and at 30kVp without any filtration denoted by “High” and “Low”, respectively are shown in Fig. 1(b,c). Both have been calibrated using the water and air regions such as to express attenuation (μ) in units [cm^−1^]. Water attenuation was calibrated to be 0.25 cm^−1^ and 3.3 cm^−1^ for the “High and “Low” scans respectively, as estimated theoretically. The grayscale in each slice image was in the range ±25% of water attenuation, to enable comparison between the two. The composition of each of the microtube is marked in Fig. 1(b). Clearly, observing each slice on its own, it is impossible to know which solution is present in each microtube. Close examination of Fig. 1(c), shows visible enhancement of the iron containing microtubes at the “Low” energy scan as compared to the “High”. It is this enhancement that enables material analysis.

Two common methods for performing material analysis are scatter plot material analysis^24^ and the related material basis decomposition^19,25,26^. Both methods can be applied in the case of K-edge imaging, as shown in Fig. 1(d–f) and (g,h), respectively. In this study, we focus on the scatter plot approach for material analysis. In the scatter plot material analysis, a scatter plot is generated for all pixels in the slice image. Each point derives its (x-y) coordinate from the attenuation values in the “High” and “Low” images, respectively, as shown in Fig. 1(d). For clarity, “point” will be used to refer to elements in the scatter plots and “pixel” to elements in the images. The scatter plot consists of clouds of points, each associated with material composition in one microtube. Increasing concentration will shift the scatter point to higher attenuation values, i.e. points will move to the top-right position in the scatter plot. The exact slope will depend on the chemical composition of the material. Slopes (α) of ${\propto }_{Fe}$ = 16.4 ± 1.6 and ${\propto }_{U}$ = 9.5 ± 0.47 were obtained for iron and uranium at this scan configuration, respectively. Material analysis capability will strongly depend on three factors. First, noise will affect the spread of points in the scatter clouds. Denoising methods that preserve resolution are typically applied to confine this spread. Second, concentration magnitude determines the distance from background along a given slope. The smaller the concentration, the more difficult the analysis becomes. Third, chemical composition determines the slope magnitude in the scatter plot. Chemically distinct materials will have largely different slopes enabling easier separation. Noise, slope magnitude and the lower bound for concentration detectability will all be strongly influenced by the selected scan configuration. The performed material analysis, was based on the mid-slope by which any point above is considered to be iron, and any point below is considered to be uranium. A calibration based on the known material concentration was performed to scale either the “High” or “Low” image, obtaining the iron material density image [mgFe/cc] shown in Fig. 1(e) in red colormap, and uranium material density image [mgU/cc] shown in Fig. 1(f) in green colormap.

Material analysis can also be done by way of material basis decomposition^19,25,26^, whereby the absorption of any material can be expressed by weighted summation of absorption of two basis material, iron and uranium in our case. A simplified approach formulates the problem as for two monoenergetic beams. Then, by solving two equations, one for each scan configuration, the density component of each basis material, is easily extracted. Specifically, the two equations are:

1 $${\mu }_{M}^{Low}={\hat{\mu }}_{Fe}^{Low}\cdot {\hat{\rho }}_{Fe}+{\hat{\mu }}_{U}^{Low}\cdot {\hat{\rho }}_{U}$$

2 $${\mu }_{M}^{High}={\hat{\mu }}_{Fe}^{High}\cdot {\hat{\rho }}_{Fe}+{\hat{\mu }}_{U}^{High}\cdot {\hat{\rho }}_{U}$$

where, ${\mu }_{M}^{Low}$ is the attenuation (or absorption interchangeably) of any material M, at “Low” energy scan configuration, ${\hat{\mu }}_{Fe}^{Low}$ is the mass attenuation coefficient of iron at “Low” scan configuration, i.e., ${\hat{\mu }}_{Fe}^{Low}=\frac{{\mu }_{Fe}^{Low}}{{\rho }_{Fe}}$ with ${\mu }_{Fe}^{Low}$ the attenuation coefficient of iron and ${\rho }_{Fe}$ is the density of iron. Similarly, ${\hat{\mu }}_{U}^{Low}$ is the mass attenuation coefficient of uranium. ${\hat{\rho }}_{Fe}$ and ${\hat{\rho }}_{U}$ are the material density components for iron and uranium, respectively, which together are used to represent material *M*. Equation (2) is similar to eq. (1) but for “High” scan configuration. As detailed in the supplemental material S2, ${\hat{\rho }}_{Fe}$, is a weighted subtraction of the “Low” image minus the “High” image and ${\hat{\rho }}_{U}$ is a weighted subtraction of the “High” image minus the “Low” image. The generated material basis decomposition images or simply material density images for iron and uranium are shown in Fig. 1(g) and (h), respectively.

**Figure 2:**
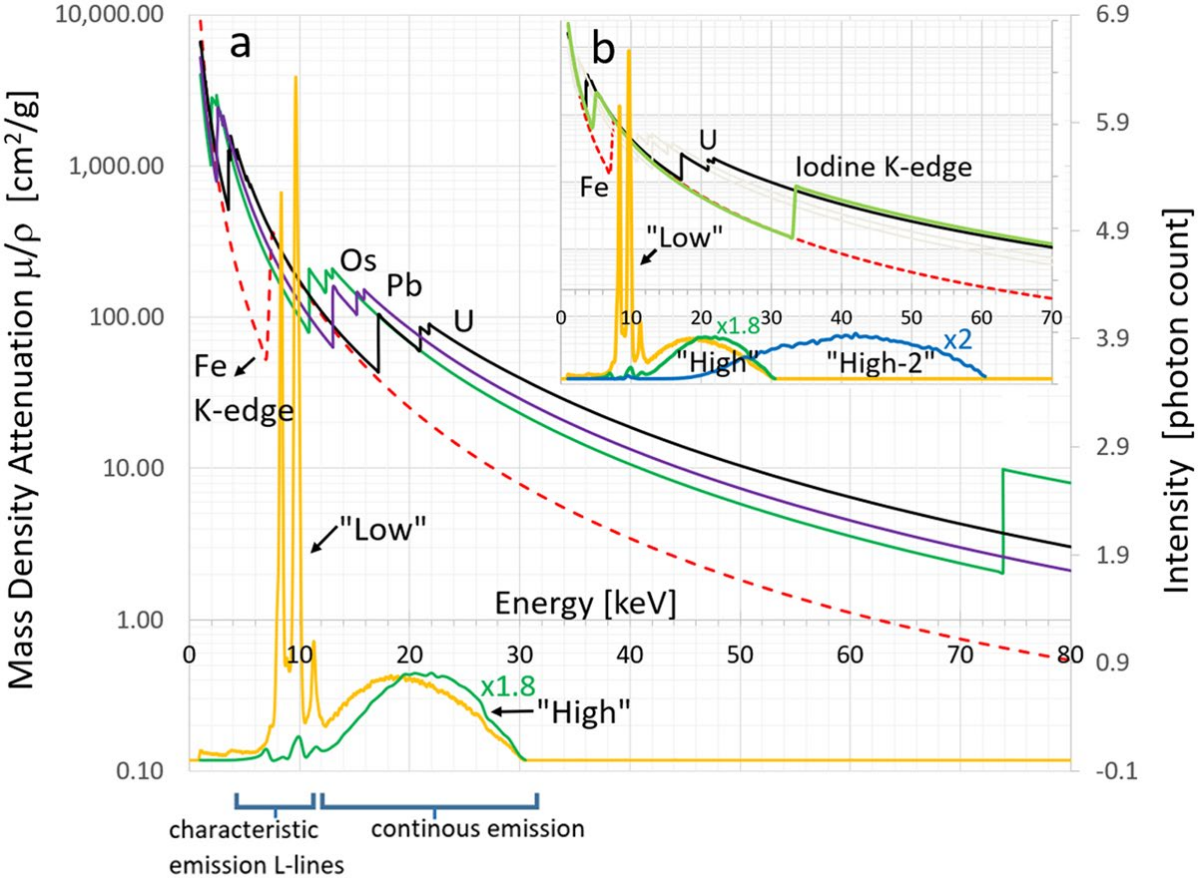
X-ray characteristic emission spectrum and mass attenuation coefficient (energy) curves for several scan configurations and elements of interest. To create a strong elemental contrast, you need both cases of enhancement and suppression of Emission-absorption overlap. Source characteristic emission L-lines and location of iron K-edge onset at lower energy are used to enhance iron absorption. (**a**) Mass density attenuation coefficient (energy) curves $\mu /\rho \,[c{m}^{2}/g]$ for osmium (Os), lead (Pb), uranium (U) and iron (Fe) are plotted in logarithmic scale on the left y-axis. Iron is in dashed curve while all the other are solid line curves with the element symbol located at the edge onset. Measured X-ray emission spectrum [photon count] are plotted in the lower part of the graph using the right y-axis, showing two scan configurations, “Low” 30 kVp unfiltered and “High” 30 kVp filtered with 25 μm thick iron foil. “High” energy spectrum was scaled x1.8. The peak tube potential can be identified by the cuff-off energy of the spectrum. (**b**) Includes, iodine mass attenuation coefficient and third scan configuration, “High-2” 60 kVp filtered with 25 μm thick tungsten foil. Some of the other curves have been fainted out for clarity. “High-2” energy spectrum was scaled x2. The two scans “High” and “High-2” are pre- and post- iodine K-edge enhancing iodine contrast - a second potential marker.

We used the measured X-ray tube spectrums and theoretical elemental mass attenuation (energy) curves from NIST XCOM database^27^, as shown in Fig. 2(a), to simulate osmium to iron absorption ratio, in order to find conditions of maximal iron absorption. We varied input spectrum peak tube potential, filtration material, filtration thickness, and accounted for detector stopping power. We found the two scan conditions with maximum and minimum absorption ratio, termed as “Low” and “High”, to be 30 kVp with no filtration and 30 kVp with 25 μm thick iron foil filtration, respectively. The terms “Low” and “High” stand for the relative value of the mean spectrum energy. Using principles of K-edge imaging^18^, we note:

1. Tungsten source characteristic emission L-lines (8.3 keV, 9.6 keV and 11.3 keV) are dominant at low tube voltage setting, e.g. at 30 kVp peak tube voltage, these account for almost 50% of the total spectrum energy being below 14 keV. The total spectrum energy being defined as the integrated photon energy,
  3 $${\int }_{0}^{\infty }N(E)EdE,N(E)-{\rm{number}}\,{\rm{of}}\,{\rm{photons}}\,{\rm{at}}\,{\rm{energy}}\,E.$$
2. Iron K-edge absorption onset (7.1 keV) is at energy just smaller than the source emission lines, resulting in good emission-absorption overlap enhancing iron absorption. The abrupt jump in iron absorption at the K-edge location is approximately 8-fold.
3. Filtered spectrum (30 kVp with 25 μm thick iron filter) characteristic emission lines are suppressed, making continuous emission dominant and shifting the energy spectrum to a higher average value, resulting in the reduction of iron absorption relative to other metals. Now, less than 9% of the total spectrum is energy below 14 keV.

**Figure 3:**
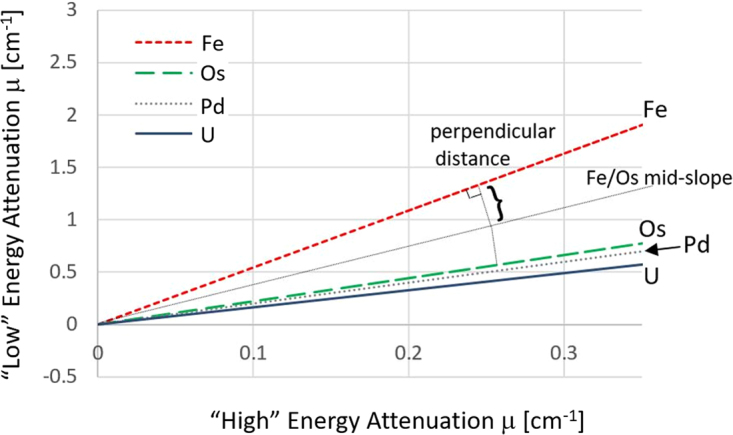
Calculated absorption scatter/slope plot of various metals present in the sample using the “Low” – (30 kVp peak tube potential and no filtration) and “High”- (30 kVp peak tube potential and 25 μm thick iron filter) scan configurations. The separation capacity is a measure of ease of separation of two elements, proportional to the depicted perpendicular distance between the two slopes. The steeper slope for iron indicated it has the potential to be separated from the other metals.

Scatter plot slopes for Fe, Pb, Os, and U using the two-scan configuration, assuming 5 um thick metal, are shown in Fig. 3. A steeper slope for iron signifies its enhancement relative to other metals due to the location of its K-edge at regions of high X-ray tube flux characteristic emission, as discussed in detail before. Based on the scatter plots we defined a quantitative separation capacity measure of any two-metal pair arranged in a distance-type chart (Table 1). The scatter capacity is defined as the perpendicular distance of mid-slope to any one of the two slopes at unit attenuation.

**Table 1:** Separation capacity for metal pairs in a distance type chart.

	^26^Fe	^76^Os	^82^Pb	^92^U
^26^Fe				
^76^Os	0.86			
^82^Pb	1.04	0.08		
^92^U	1.45	0.29	0.19	

The separation capacity is defined as the perpendicular distance of mid-slope to any one of the two slopes at unit attenuation, depicted in Fig. 3. The larger the separation capacity value, the easier it will be separating the two elements. The iron containing pairs had considerably larger separation capacity than those expected between pairs containing both heavy metals.

The higher the value, the stronger is the separation capability. It is evident that iron has the potential to be separated at these conditions from the other metal elements. On the other hand, for example, separating osmium and lead would be a very difficult task. As tube output increases with voltage improving signal to noise ratio, we calculated the impact of increasing the tube voltage for the “High” scan, keeping existing filtration. At 60 kVp peak tube potential for which power is increased by a factor x2.5, we found the calculated slope and separation capacity to be within 2% of previously calculated values. And so, we performed the following two experimental scans, “Low” energy scan at 30 kVp with no filtration and a “High” energy scan at 60 kVp with 25 mm thick iron filter.

**Figure 4:**
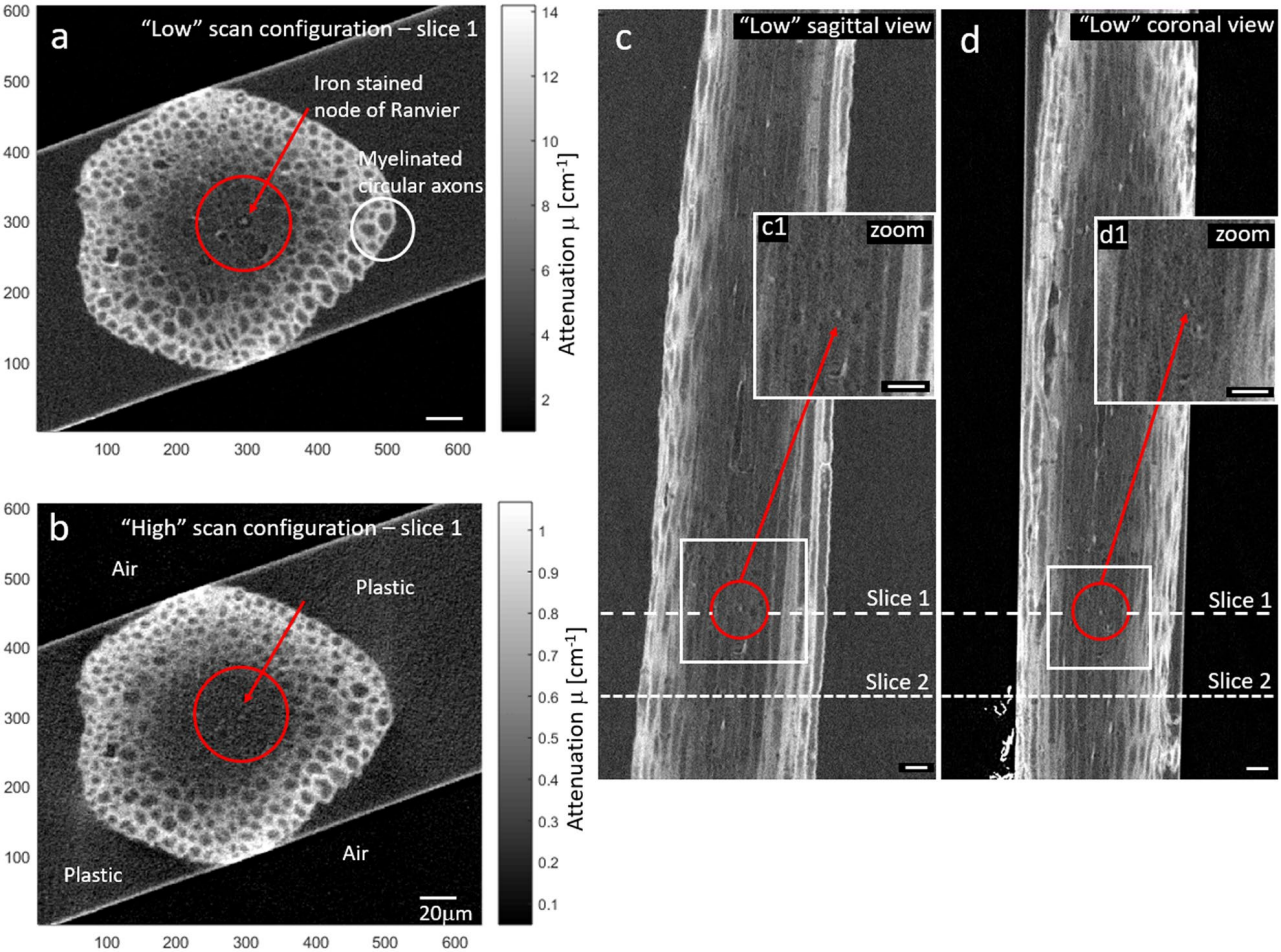
Experimental XRM images of a mouse spinal root, stained with iron and osmium. (**a**,**b**) “Low” and “High” energy cross sectional images of spinal root, respectively. Red arrow points to location of node of Ranvier at center of red circle, specifically stained with iron. The circular structures are myelin sheaths stained with osmium. Air and plastic regions were used to calibrate. To ease comparison each image grayscale was set in the range [min, max] = [0, 4x (plastic value)]. In (**b**), air and plastic regions have been labeled. (**c**,**d**) Sagittal and coronal cross sections. Both images (**a**,**b**) are cross sections located at position of dashed line labeled slice 1. Images of cross section labeled slice 2 will be discussed in next figure. Enlargements of boxed regions around the marked node (c1-d1) are inserted in each. Scale bars are all 20 μm.

Next, we investigated a sample of spinal root extracted from the peripheral nervous system of a mouse, as shown in Fig. 4. The sample has been stained with iron, which is known to specifically target nodes of Ranvier^28–30^, and then subsequently stained with 1.0% osmium tetroxide^31^ as detailed in the supplemental material S3. The iron staining protocol was originally used to investigate axonal membrane structure, targeting the nodal axolemma with iron. Osmium is known to dominantly stain the myelin sheaths of the neuronal axon.

Figure 4(a,b) are the “Low” and “High” slice images of the spinal root cross-section. Images have been calibrated to express attenuation in units of [cm^−1^]. The packed bright circular structures are myelin sheaths surrounding axons, heavily stained with osmium. As can be seen, staining is largest at the root periphery and reduced towards the center due to limited reagent accessibility. Additional sagittal and coronal views (perpendicular cross sections of the “Low” scan) are shown in Fig. 4(c,d). Iron-stained nodes of Ranvier appear as bright spots surrounded by a black rim (Fig. 4(a)). The nodes are situated at points along axons where there is an absence of myelin, explaining their characteristic appearance, i.e. a bright spot due to iron surrounded by a dark ring due to the absence of osmium. Other bright spots throughout the volume were found to be associated with iron staining of Schwann cell nuclear nucleus.

**Figure 5:**
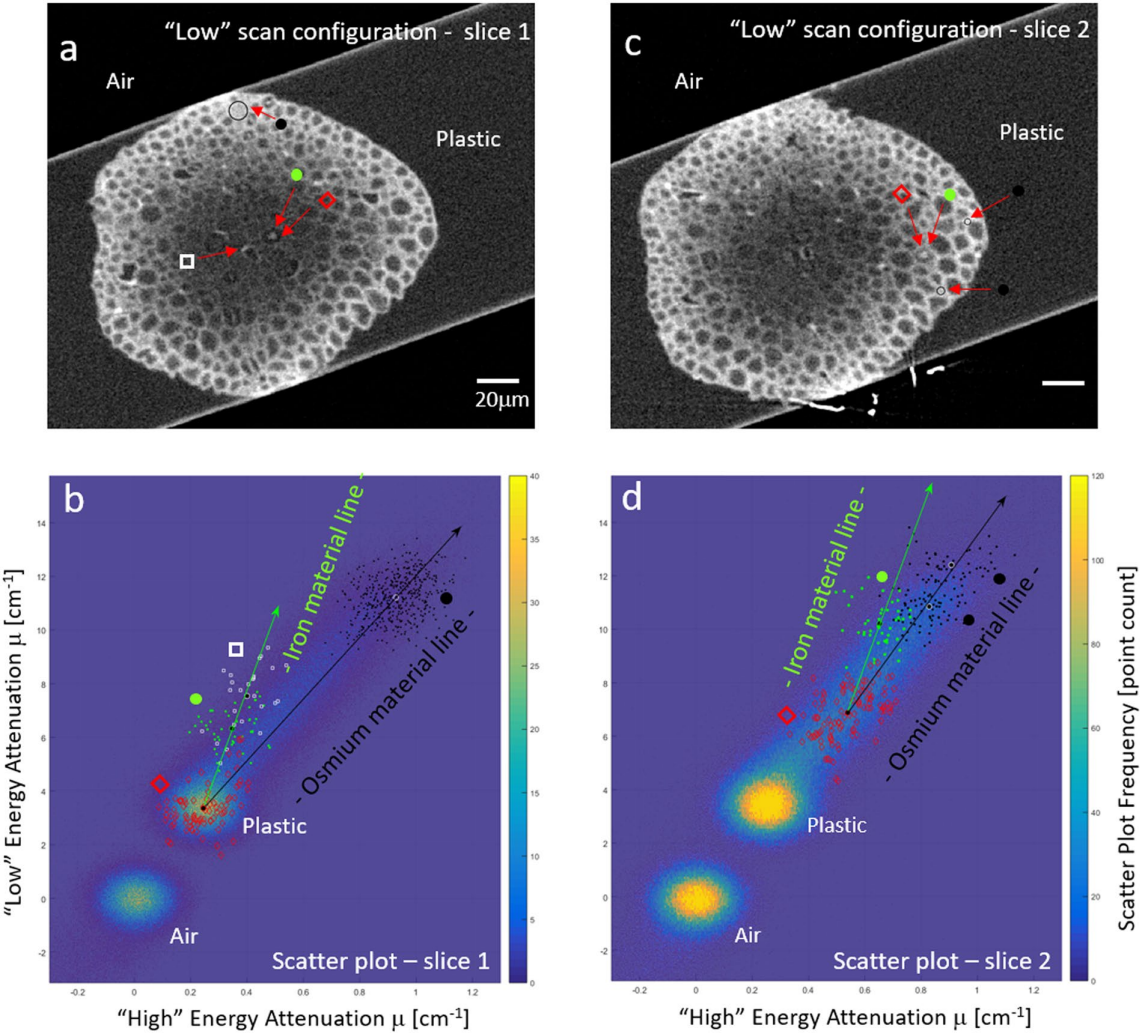
Scatter plots of the spinal root sample. Scatter plots (**b**,**d**) were generated by mapping each pixel by its “High” and “Low” attenuation value. Regions within the scatter plot can be traced to specific regions in the slice images shown in (**a**,**c**). Slice 1 (**a**) exemplifies a node in a low staining background. Two large point densities in the scatter plot are associated with air and plastic regions. A node (*green circle*) and another bright spot (*white square*) project in the scatter plot at a slope characteristic of iron, as seen when individual pixels associated with ROI are marked similarly in the scatter plot. Background locations with no prominent staining (*red diamonds*) were taken from the circular dark ring around the node and are associated with plastic. Myelin (*black circles*) ROI project along a line with a slope characteristic for osmium. Slice 2 (**c**) exemplifies a node in a region with considerable osmium staining, and so the iron is projected in the scatter plot (**d**) from a location already exhibiting some given osmium concentration.

To analyze material composition within the sample, we generated scatter plots from the “High” and “Low” images of the spinal root, as shown in Fig. 5. All pixels in the slice image, shown in Fig. 5(a) were used to generate the scatter plot shown in Fig. 5(b). Once the points crowd and overlap, we look now at density of points in units of point count. Air and plastic are both highly abundant and generate high count regions marked as “Air” and “Plastic”.

As expected for calibrated images the air region is centered at attenuation value (0,0). Pixels associated with other ROI within the image, as marked in Fig. 5a, can be mapped within the attenuation scatter plot. For clarity we show only the “Low” image, as it had stronger iron contrast and better signal to noise. As the spinal root center is only lightly stained with osmium, any iron present here will build primarily from background, which is plastic as shown in Fig. 5(b). Osmium regions populate the space in the scatter plots extending from plastic to high osmium concentration. As shown in Fig. 5(c), image slice 2 exhibits a node closer to the spinal root periphery, containing higher osmium staining. As expected, the iron is co-mingled with osmium, and yet it has a slope characteristic of iron. Thus, it is possible to distinguish ROI containing iron even in the presence of moderate levels of osmium. Location of slice 1 and 2 can be seen in Fig. 4(c,d) marked by the dashed lines.

**Figure 6:**
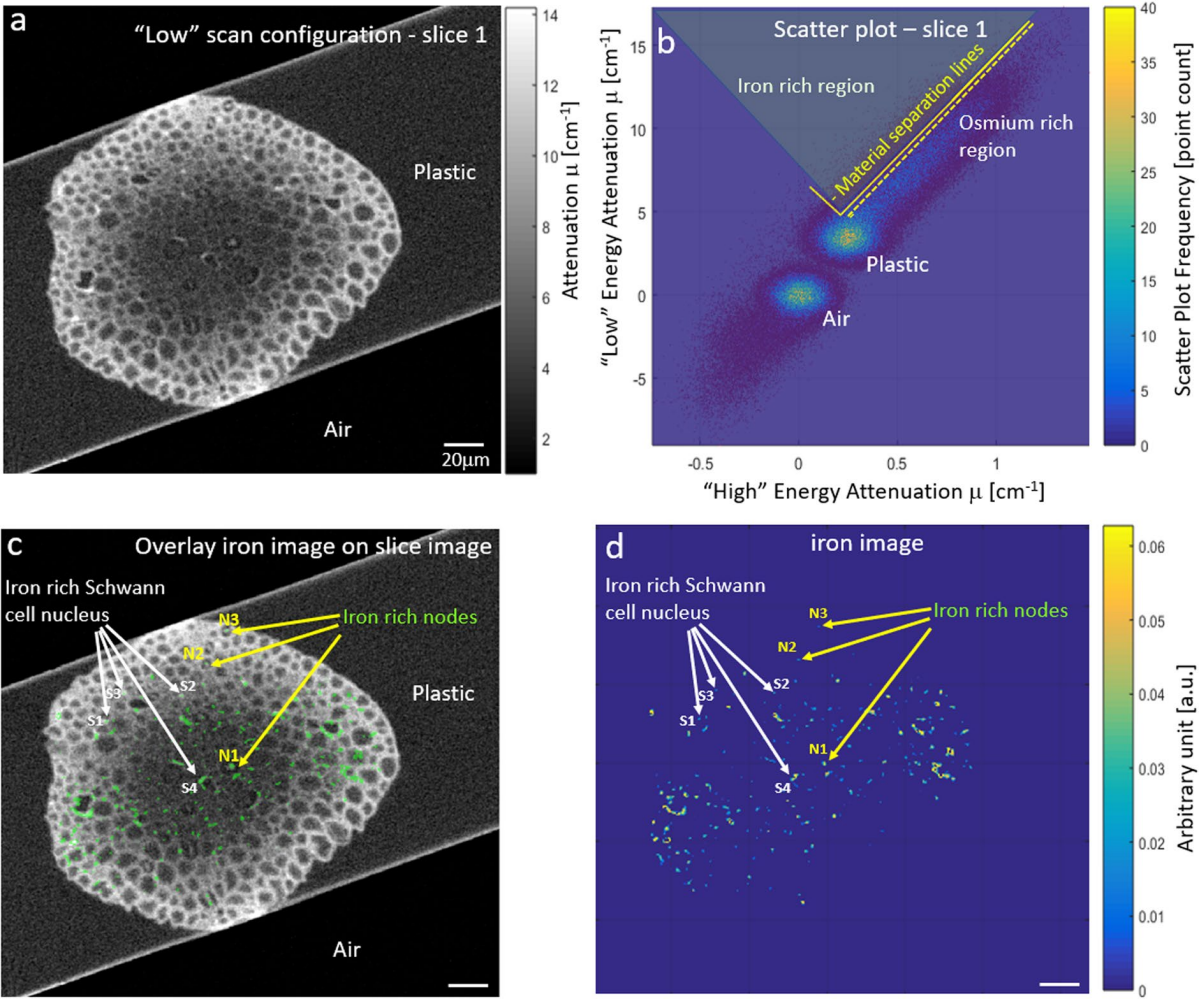
Scatter plot was used to automatically segment the iron rich nodes of Ranvier regions. Other regions stained with iron were found out to be iron staining of the Schwann cell nucleus. (**a**) Slice image (**b**) Scatter plot allowing material analysis using shown material separation lines. (**c**) Overlay of Material analysis iron image (green) on original slice image. (**d**) Iron image proportional to iron density, in arbitrary units. All scale bars are 20 μm.

We looked at the average line slopes (α) generated by iron and osmium material. For slice 1 and 2 we measured $({\alpha }_{Fe}^{slice1}$ = 28 ± 21, ${\alpha }_{Os}^{slice1}$ = 11.5 ± 3.7) and $({\alpha }_{Fe}^{slice2}$ = 27 ± 16, ${\alpha }_{Os}^{slice2}$ = 14.5 ± 5.7), respectively. Though the spread of points can be large, looking at the average value aids the analysis. It is the ability to identify different slopes (i.e. material lines) within the same sample, for iron and osmium in this case, that enables material analysis as shown in Fig. 6.

To locate the iron-rich regions throughout a slice image, we applied a material separation line to the scatter plot, as shown in Fig. 6(b). To overcome noise and refine the material analysis, we used a scheme that not only looks at the location within the scatter plot but also considers the character of neighboring pixels, to improve the material analysis, as described below.

1. Points above the material separation line, i.e. the iron rich region, are initially selected to be iron pixels.
2. For each selected point, we examine the points associated with its neighboring pixels in the slice image within a specified radius (e.g. radius 3 pixel). If the centroid of these points is within a given distance to the original separation line (which can be tuned) this confirms the classification of the initial pixel as iron-rich and suggests nearest neighbors as possible iron-rich candidates as well. If not, i.e. it is too close to the osmium-rich region, it is removed.
3. In an iterative manner, the added pixels are checked with the aid of their neighboring pixels to decide if they should be classified as iron-rich.

Nodes of Ranvier can be clearly identified as iron-rich in images analyzed by this method, as shown in Fig. 6(c) and marked N1-3. Surprisingly, other iron-rich regions with a circular morphology were also detected, as shown in Fig. 6(c) and marked S1-4. Examination of these ROI by transmission EM (TEM) revealed these structures to be related to Schwann cell nucleus. The iron image shown in Fig. 6(d), is proportional to iron density and was calculated from the distance of iron points to the material separation line, yet not quantified due to lack of calibration.

**Figure 7:**
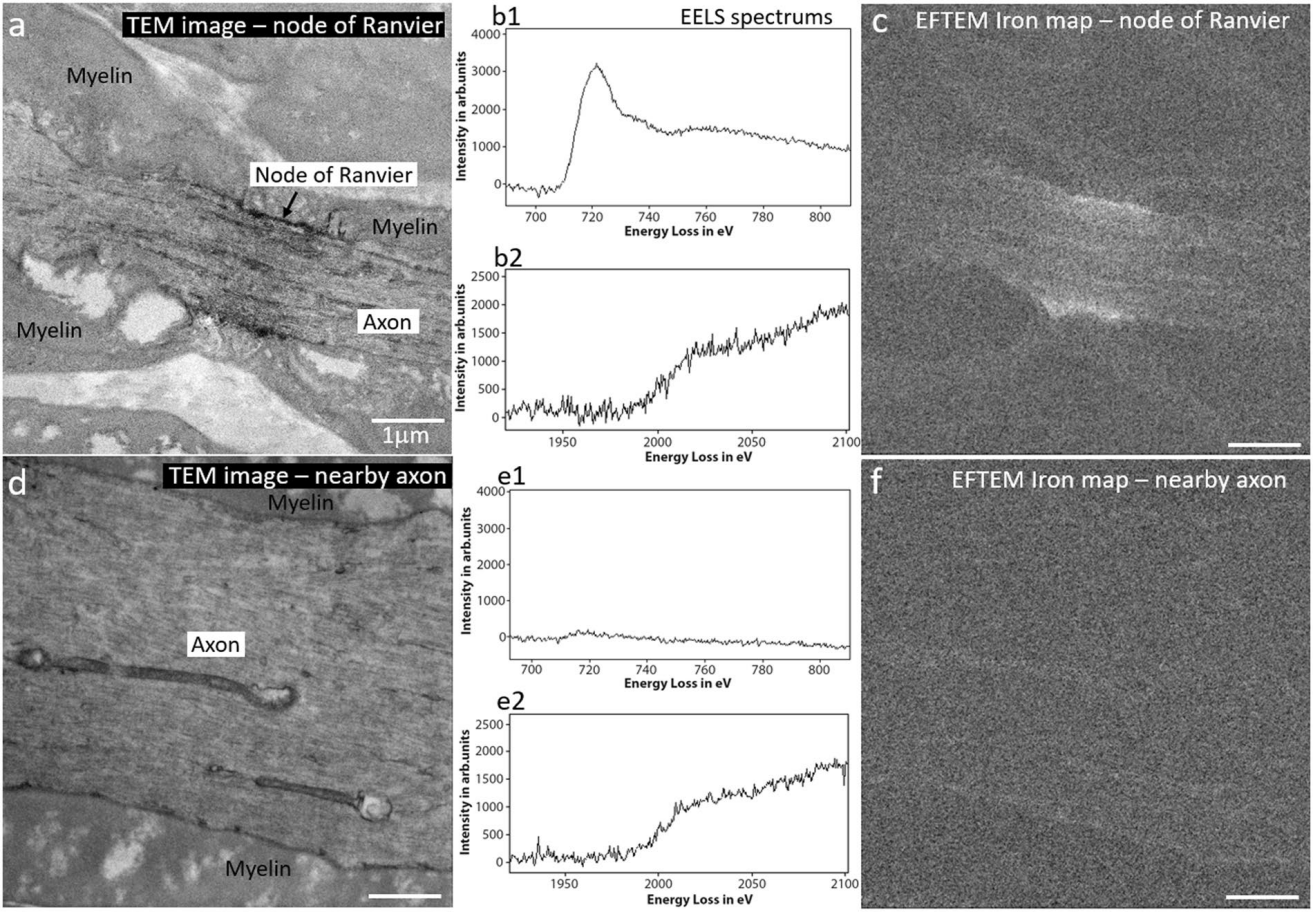
TEM and EELS measurements at the node of Ranvier and at a nearby control region. (**a**) TEM image showing staining of the node. EELS spectrum taken at the node showing strong presence of iron (b1) and osmium (b2) for which edge onset occurs at 708 eV and 1960eV, respectively. (**c**) EFTEM iron map confirms iron presence at the node (**d**) TEM of a control region showing a nearby axon of larger diameter. EELS spectrum taken at the control region showing the absence of iron (e1) yet presence of osmium at similar levels seen at the node (e2). (**f**) EFTEM iron map showing signal at noise level indicating the absence of iron in the control region. Scale bar for all images is 1 μm.

We used a Transmission Electron Microscope (TEM) equipped with an in-column Electron Energy-Loss Spectrometer (EELS), to further validate our findings and to check that iron was indeed localized to the nodes of Ranvier and was not present elsewhere in the sample (Fig. 7). The TEM image of the nodes of Ranvier in a central spinal root region reveals dense staining (Fig. 7a) which is confirmed to be iron from the iron map obtained by Energy-Filtered TEM (EFTEM) mapping (Fig. 7c). The EELS spectra taken at the node reveals presence of both iron (Fig. 7b1) and osmium (Fig. 7b2) edge onset of which occurs at 708 eV (L_2,3_ Edge) and 1960 eV (M_4,5_ Edge) respectively^32^. Additionally, we performed EELS measurements at a control region in the same sample i.e. a region away from the node of Ranvier. (Fig. 7d). As can be seen from the spectrum (Fig. 7e1 and e2), this region had approximately the same amount of osmium but only negligible amount of iron in comparison with the node of Ranvier. The absence of iron in the control region, is further corroborated by the EFTEM iron map (Fig. 7f), which shows only noise. TEM images also revealed that the circular-shaped objects observed in the X-ray, were Schwann cells nuclei. The EELS spectrum taken at these locations confirmed the presence of iron (data not shown). Schwann cells are responsible for producing the myelin sheath around the neuronal axons and staining of the nuclear membrane and heterochromatin were visible.

## Conclusions

We show that we can use the methods developed and described here to identify iron-rich regions in a sample stained with other elements (heavy metals) prepared for X-ray microtomography and EM imaging. Applying a two-scan configuration, iron absorption was selectively either enhanced or suppressed, providing strong contrast due to differences in X-ray source spectrum in the two cases. Good overlap of source characteristic emission lines and iron K-edge absorption was needed to achieve this contrast.

The results demonstrated here for iron can be extended to other elements in the future, by using non-tungsten X-ray sources, e.g. chrome or nickel target X-ray sources, and checking overlap of elements absorption edges. For example, it can be expected that cerium (6.6 keV L1-edge) will present similar results to iron (6.1 keV K-edge) with current tungsten source.

Some limitation is associated with the maximum sample size for which reasonable contrast can still be achieved. In this work, we imaged biological samples of physical size in the [mm] scale looking at features at sub-micron resolution. Too thick of a sample will result in X-ray beam filtration, depleting primarily the low energy part of the spectrum in a process called “beam hardening”. As a result, the source characteristic emission lines will be suppressed, decreasing the iron absorption and resulting in smaller iron contrast. We found samples with path lengths of approximately 1 mm are free of this limitation. For the same reason, filtered X-ray sources used in medical imaging (used to reduce skin dose) will not exhibit strong iron absorption, as shown here due to complete depletion of the X-ray spectrum below 30 keV.

A second elemental detection can potentially be achieved by adding a third scan configuration. Targeting iodine, a third post-edge scan configuration (iodine K-edge 33.2 keV), labelled “High-2” together with previous “High” scan will achieve a pre- post- iodine K-edge pair achieving strong iodine contrast. Specifically, source 60 kVp peak tube potential filtered with 25 μm thick tungsten foil will have good iodine absorption and source spectrum overlap to achieve iodine enhancement. The third scan configuration “High-2” and iodine mass attenuation (energy) curve are shown in Fig. 2(b). The addition of a second element detection to the shown iron detection, to be included in future work, will potentially achieve multi-color X-ray imaging similar to what was demonstrated in EM using lanthanide labels and EELS^5^.

In summary, we have shown a method that uses XRM with a polychromatic source to detect a specific element within a heavy metal stained biological sample, by maximizing X-ray source emission and elemental absorption overlap. Scatter plots where shown to be instrumental in identifying regions of distinct chemical signature, enabling automated material analysis. Using the scheme presented, a second element detection could be pursued. Correlating the target metals (e.g., Fe) to fluorescent genetic-probes, similar to that demonstrated in multicolor electron microscopy^5^, will provide important large volume functional localization information.

## Methods

### X-ray microtomography

XRM was performed on a Versa 510, (Carl Zeiss X-ray Microscopy, Pleasanton, CA 94588). The Versa 510 is equipped with a polychromatic tungsten source, tunable in the voltage range 30–160 kVp. Scan configuration: “Low” −30 kVp peak tube potential without filtration, exposure time 20–90 sec. “High”− 60 kVp peak tube potential, filtration 25 μm thick iron filter, exposure time 10–120 sec. Common scan conditions: binning 1, source-rotation axis distance 7.5 mm, detector-rotation axis distance 5 mm, pixel size 0.394μm, optical magnification x20, rotation angle −180° to +180°, number of views 3201. Iron filtration used a 0.001”x1”x2” foil placed at the X-ray tube exit slit. The foil was of 3N5 purity and sold by ESPI Metals, Ashland, OR 97520.

### Staining protocol

Staining protocol is provided as Supplementary Material S3.

### Simulation

Material mass attenuation (energy) curves for the different elements were taken from NIST – XCOM database^27^ for energy range 1–160 keV at intervals 0.5 keV. The X-ray spectrums were measured experimentally using AMETEK Material Analysis Division X-123CdTe X-ray Spectrometer, Amptek, Inc. Bedford, MA 01730. Typical resolution at FWHM was specified to be <1.2 keV at 120 keV. Experimentally measured X-ray spectrums for 30 kVp with and without 25 um thick iron filter, and 60 kVp with 25 um thick tungsten filter were presented in Fig. 2.

### TEM and EELS measurements

The TEM imaging was performed with a JEOL JEM-Z3200EF operating at 300 kV. This TEM has an Electron Energy-Loss Spectrometer in the form of an in-column Omega filter. All images, spectra and maps were acquired using Digital Micrograph software on a Gatan (Pleasanton, USA) US 4000 CCD camera. Prior to imaging, the sample was pre-irradiated at a low magnification for about 30 min to minimize the effects of contamination^33^.

The Iron spectra were acquired for 5 sec exposures. The EELS signals drop progressively with increasing energy loss and typically reaching negligible levels around and above 2000 eV^34^. The energy loss edge onset for osmium is at 1960 eV making it particularly challenging to obtain spectra in this energy domain. We overcame this problem by binning the camera pixels to 4 and using the microscope in diffraction mode, so to boost the signal-to-noise. The Osmium spectra were acquired for 360 sec exposures. The EFTEM images for computing the iron maps were acquired for 120 sec exposures with the CCD bin by 4 pixels.

### Data Availability

XRM image data generated and analyzed in this study will be available to download from the Cell Centered Database and Cell Image Library under the link, http://cellimagelibrary.org/groups/50351.

## Electronic supplementary material

supplemental material
