# Supplementary material for: Iron-specific Signal Separation from within Heavy Metal Stained Biological Samples Using X-Ray Microtomography with Polychromatic Source and Energy-Integrating Detectors
Source: Sci Rep. 2018 May 15;8:7553. doi: 10.1038/s41598-018-25099-z (PMC5953933; doi:10.1038/s41598-018-25099-z)
Supplement: Supplementary file 1 — supplemental material [file 41598_2018_25099_MOESM1_ESM.docx]

**Iron-specific Signal Separation from within Heavy Metal Stained Biological Samples Using X-Ray Microtomography with Polychromatic Source and Energy-Integrating Detectors**

Tsvi Katchalski,^1*^ Tom Case,^2^ Keun-young Kim,^1^ Ranjan Ramachandra,^1^ Eric A. Bushong,^1^ Thomas J. Deerinck,^1^ Matthias G. Haberl,^1^ Mason R. Mackey,^1^ Steven Peltier,^1^ Guillaume A. Castillon,^1^ Nobuko Fujikawa,^2^ Albert R. Lawrence,^1^ and Mark H. Ellisman^1,3^

^1^National Center for Microscopy and Imaging Research (NCMIR), University of California San Diego, 9500 Gilman Dr. MC 0608, La Jolla, CA 92093-0608, USA

^2^*Carl Zeiss X-Ray Microscopy, 4385 Hopyard Road, Suite 100. Pleasanton, CA 94588, USA*

*^3^Departments of Neurosciences and Bioengineering, University of California San Diego, 9500 Gilman Dr. MC 0608, La Jolla, CA 92093-0608, USA*

^*^*Corresponding authors:* [*tkatchalski@ucsd.edu*](mailto:tkatchalski@ucsd.edu)*; mellisman@ucsd.edu;*

**Supplemental Material**

**Supplemental material S1 – Iron and uranium aqueous solution phantom**

The various aqueous solutions were contained in polyimide tubing (Cole-Palmer, IL) at tube inner diameter (ID) 0.02”. The solutions were forced into the tube using a micropipette. The tubes were then sealed by dipping in “plumber’s glue” (Christy’s Blue Glue). We prepared the solutions from, Iron(III) chloride hexahydrate (Sigma-Aldrich) and uranyl acetate (Electron Microscopy Science, PA) dissolved in a doubly distilled water (DDW). Final concentrations were noted in [%] by weight.

**Supplemental Material S2 – Material basis decomposition equations**

Estimates for Fe and U (or osmium for that matter) material basis images can be obtained from the approximate equations given below^19,25,26^.

Starting from the general equation describing the absorption mass density of any material - M, $\frac{\mu_{M}}{\rho_{M}}$ as a sum of contributions of the basis material,

$\frac{\mu_{M}}{\rho_{M}}=a_{1}\cdot\frac{\mu_{Fe}}{\rho_{Fe}}+a_{2}\cdot\frac{\mu_{U}}{\rho_{U}}$

and rewriting to obtain,

$\mu_{M}=a_{1}\cdot\frac{\mu_{Fe}}{\rho_{Fe}}\rho_{M}+a_{2}\cdot\frac{\mu_{U}}{\rho_{U}}\rho_{M}$

and re-symbolizing, the absorption mass density, $\frac{\mu}{\rho}\to\hat{\mu}$and $a_{1}\cdot\rho_{M}\to\hat{\rho}_{Fe}$, $a_{2}\cdot\rho_{M}\to\hat{\rho}_{U}$ to obtain,

$\mu_{M}=\hat{\mu}_{Fe}\cdot\hat{\rho}_{Fe}+\hat{\mu}_{U}\cdot\hat{\rho}_{U}$ .

And so, the two equations formulating the decomposition describing the absorption of material -*M*, at any location:

1. $\mu_{M}^{Low}=\hat{\mu}_{Fe}^{Low}\cdot\hat{\rho}_{Fe}+\hat{\mu}_{U}^{Low}\cdot\hat{\rho}_{U}$
2. $\mu_{M}^{High}=\hat{\mu}_{Fe}^{High}\cdot\hat{\rho}_{Fe}+\hat{\mu}_{U}^{High}\cdot\hat{\rho}_{U}$

where, $\mu_{M}^{Low}$ is the measurable attenuation (or absorption interchangeably) of any material *M*, at “Low” energy scan configuration, $\hat{\mu}_{Fe}^{Low}$ is the mass attenuation coefficient of iron at “Low” scan configuration, i.e., $\hat{\mu}_{Fe}^{Low}=\frac{\mu_{Fe}^{Low}}{\rho_{Fe}}$ with $\mu_{Fe}^{Low}$ the attenuation coefficient of iron and $\rho_{Fe}$ is the density of iron. Similarly, $\hat{\mu}_{U}^{Low}$ is the mass attenuation coefficient of uranium. $\hat{\rho}_{Fe}$ and $\hat{\rho}_{U}$ are the density component of material iron or uranium representing together material *M*. Same notation applies when replacing “Low” with “High”.

Solving, we arrive at equations for the material density component of iron and uranium:

1. $\hat{\rho}_{Fe}=\frac{\hat{\mu}_{U}^{High}}{\hat{\mu}_{U}^{High}\cdot\hat{\mu}_{Fe}^{Low}-\hat{\mu}_{U}^{Low}\cdot\hat{\mu}_{Fe}^{High}}\left\{ \mu_{M}^{Low}-{\frac{\hat{\mu}_{U}^{Low}}{\hat{\mu}_{U}^{High}}\mu}_{M}^{High} \right\}$

Make note that the iron density component is proportional to “Low” image minus a scaled “High” image. The scaling $\frac{\hat{\mu}_{U}^{Low}}{\hat{\mu}_{U}^{High}}$ is the material slope of uranium in the scatter plot.

1. $\hat{\rho}_{U}=\frac{\hat{\mu}_{Fe}^{Low}}{\hat{\mu}_{U}^{High}\cdot\hat{\mu}_{Fe}^{Low}-\hat{\mu}_{U}^{Low}\cdot\hat{\mu}_{Fe}^{High}}\left\{ \mu_{M}^{High}-{\frac{\hat{\mu}_{Fe}^{High}}{\hat{\mu}_{Fe}^{Low}}\mu}_{M}^{Low} \right\}$

Similarly make note that this time, the uranium density component is proportional to “High” image minus a scaled “Low” image. The scaling $\frac{\hat{\mu}_{Fe}^{Low}}{\hat{\mu}_{Fe}^{High}}$ is the material slope of iron in the scatter plot.

Equations (3-4) were used to generate the material basis decomposition images using basis material measured values of attenuation.

**Supplemental material S3 – Mouse spinal cord perfusion and staining protocol using ferric chloride and ferrocyanide for X-ray electron microscopy**

1. Fix with 2.5% glutaraldehyde and 2% paraformaldehyde
2. Postfix for 1 hour
3. Rinse in 0.15M sodium cacodylate buffer 5* x 5 minutes on ice
4. Postfix in 1% osmium tetroxide for 1 hour
5. Rinse in DDW 5 x 3 minutes
6. Stain in 0.01M FeCl3 for 1 hour rotate at RT
7. Rinse in DDW 5 x 3 minutes
8. Stain in 1% K4Fe(CN)6-3H20 (pH 2) for 20 minutes rotate at RT
9. Rinse in DDW 5 x 3 minutes
10. Dehydrate in an EOTH series on ice 3 minutes each
11. Dehydrate in 100% ETOH 2 x 5 minutes at RT
12. Into 1:1 ETOH:Durcupan overnight
13. Into 100% Durcupan 3 x 2 hours
14. Place in oven for 48 hours

* The notation used throughout the steps, such as, “5 x 5 minutes…” is short for: repeat, i.e. repeat five times for five minutes…
